# *Pseudomonas aeruginosa* reference strains PAO1 and PA14: A genomic, phenotypic, and therapeutic review

**DOI:** 10.3389/fmicb.2022.1023523

**Published:** 2022-10-13

**Authors:** Amber Grace, Rajnish Sahu, Donald R. Owen, Vida A. Dennis

**Affiliations:** ^1^Department of Biological Sciences, Alabama State University, Montgomery, AL, United States; ^2^Owen Biosciences Inc., Baton Rouge, LA, United States

**Keywords:** *Pseudomonas aeruginosa*, PAO1, PA14, *Pseudomonas* genome, ESKAPE pathogens, clinical relevance of *Pseudomonas aeruginosa* strains, *Pseudomonas aeruginosa* reference strains

## Abstract

*Pseudomonas aeruginosa* is a ubiquitous, motile, gram-negative bacterium that has been recently identified as a multi-drug resistant pathogen in critical need of novel therapeutics. Of the approximately 5,000 strains, PAO1 and PA14 are common laboratory reference strains, modeling moderately and hyper-virulent phenotypes, respectively. PAO1 and PA14 have been instrumental in facilitating the discovery of novel drug targets, testing novel therapeutics, and supplying critical genomic information on the bacterium. While the two strains have contributed to a wide breadth of knowledge on the natural behaviors and therapeutic susceptibilities of *P. aeruginosa*, they have demonstrated significant deviations from observations in human infections. Many of these deviations are related to experimental inconsistencies in laboratory strain environment that complicate and, at times, terminate translation from laboratory results to clinical applications. This review aims to provide a comparative analysis of the two strains and potential methods to improve their clinical relevance.

## Introduction

*Pseudomonas aeruginosa* is a gram-negative, opportunistic bacterium that presents critical challenges to immunocompromised hosts (Wilson and Pandey, 2022). The metabolically versatile and antibiotic-resistant nature of the pathogen contributes to increased mortality in cystic fibrosis (CF) patients, burn victims, and those with malignancy or on mechanical ventilation (Lund-Palau et al., 2016). *P. aerguinosa* has become highly resistant to traditional disinfectants and antibiotics, thus making it difficult to remove from hospital settings and to treat in infected patients (Stover et al., 2000; Hemati et al., 2020; Langendonk et al., 2021). The antibiotic resistant nature of the bacterium is even more pronounced in infections consisting of biofilms, which the pathogen readily forms (Ciofu and Tolker-Nielsen, 2019). Such resilience against available therapeutics led the World Health Organization (WHO) to classify *P. aeruginosa* as one of the ESKAPE (*Enterococcus faecium*, *Staphylococcus aureus*, *Klebsiella pneumoniae*, *Acinetobacter baumannii*, *Pseudomonas aeruginosa*, and *Enterobacter species*) pathogens in critical need of novel therapeutics (De Oliveira et al., 2020).

*Pseudomonas aeruginosa* PAO1 and PA14 have emerged as two of the most frequently employed strains to assess novel therapeutics and to understand the biology of the bacterium. PAO1 strain is a derivative of the initial PAO isolate, or “*P. aeruginosa* strain 1,” which, in 1954, was obtained from a wound in Melbourne, Australia. The PAO1 strain commonly used today results from a spontaneous mutation that conferred chloramphelicol resistance to the original PAO isolate (Chandler et al., 2019). PAO1 was the first strain to have its genome completely sequenced, and is most commonly used in the analyses of *P. aeruginosa* genetics, physiology, and metabolism (Chandler et al., 2019). Such broad use has, however, created opportunities for mutational events to yield numerous PAO1 sub-lines with various phenotypic and genetic differences (Chandler et al., 2019).

PA14, or UCBPP-PA14, is a burn wound isolate and a much more virulent pathogen in plants and animals (Mathee, 2018). PA14 was one of the strains isolated from burn wound patients at a hospital in Pennsylvania. It subsequently became part of a collection in the University of California Berkeley Plant Pathology laboratory, hence the abbreviation “UCBPP.” PA14 research began as an investigation of the link between plant pathogens and human infections and has now developed into a highly preferred model for virulent *P. aeruginosa* infections and pathogenicity research.

Both PAO1 and PA14 strains have demonstrated infectious ability in plants, vertebrates, and invertebrates, with PA14 infecting a greater portion of hosts within these categories (Mikkelsen et al., 2011). Although PA14 is the more virulent strain, it displays a high level of genomic conservation with that of PAO1 (Mikkelsen et al., 2011). On the genomic level, PA14 possesses two pathogenicity islands, which carry virulence-associated genes that are not present in the genome of PAO1 (Mikkelsen et al., 2011).

Use of the PAO1 and PA14 laboratory reference strains has provided a means for numerous researchers to produce consistent data for *P. aeruginosa* (Chandler et al., 2019). However, there have recently been calls to replace the use of reference strains with panels of diverse clinical isolates (Freschi et al., 2018). While in some scenarios such an option is ideal, such as studying *P. aeruginosa* diversity that develops in CF patients, there are many other experimental scenarios where clinical strains may not be significantly more beneficial or feasible (Freschi et al., 2018). As *P. aeruginosa* demonstrates high plasticity and environmental adaptability, any experiment that requires transitioning clinical isolates from clinical settings to laboratory environments introduces a number of genetic and phenotypic changes for which the researcher must now account (Migliorini et al., 2019). Additionally, the genetic makeup of clinical isolates can vary from patient to patient, potentially complicating the wide application of therapeutic results (Rees et al., 2019; Scheffler et al., 2022).

The two most widely used PAO1 and PA14 laboratory strains have been fully sequenced, are readily available, and have been meticulously examined for expected behaviors. While genetic mutations and phenotypic variations have appeared due to inconsistencies in laboratory maintenance, proposed methods do exist for reducing genomic and phenotypic variability (Chandler et al., 2019). Additionally, clinical relevance can be improved by ensuring that the laboratory environment for the reference strain mimics the clinical environment of interest as closely as possible (Cornforth et al., 2020; Manzo et al., 2021; Yung et al., 2021). This review will highlight the similarities and differences that should be expected when utilizing PAO1 and PA14 experimentally, as well as methods for improving their clinical relevance.

## PAO1 and PA14 genomic differences

### Core and accessory genomes

Of the bacterial genomes sequenced to date, *P. aeruginosa* has one of the largest, with approximately 7 million base pairs (Mbp) representing the higher end of the genome size (Poulsen et al., 2019). All *P. aeruginosa* strains possess a highly conserved core genome (accounting for approximately 90% of the total genome) and an accessory genome (Klockgether et al., 2011; Kandasamy et al., 2020; de Sousa et al., 2021). The determined number of core genes has varied between studies due to variations in gene annotations, strain sets, and core gene definitions. Nevertheless, Poulsen et al. recently determined that 5,109 protein-coding genes comprised the core genome of all *P. aeruginosa* strains (Ozer et al., 2014; Valot et al., 2015; Poulsen et al., 2019). Nine strains—PA14 and eight clinical strains from five infection sources (wound, urine, respiratory, ocular, and blood)—were grown in five media types [LB, minimal M9 laboratory media, fetal bovine serum (FBS), synthetic cystic fibrosis sputum (SCFM), and urine]. This comprehensive evaluation yielded a core genome assessment that should be consistent regardless of bacterial environment or infection source. Moreover, the essential core genome, calculated at 321 genes, yielded a comprehensive list of potential therapeutic targets that should be effective across *P. aeruginosa* strains and in varying environments. Of the essential core genes, approximately 41% produce cytosolic proteins involved in metabolism; 37% are involved in DNA replication, transcription, or translation; 4% produce cytoplasmic, periplasmic, and outer membrane proteins involved in cell structure and division; 3.7% produce cytoplasmic, periplasmic, and outer membrane proteins involved in metabolism; and 8% yield cytoplasmic, periplasmic, and outer membrane proteins that function as transporters/chaperones. Uncharacterized genes comprised another 3.7%. The exhaustive list of the above gene names has been reported (Poulsen et al., 2019).

The *P. aeruginosa* accessory genome holds significant therapeutic interest due to its ability to acquire genes that improve virulence, antibiotic resistance, and general bacterial fitness through horizontal gene transfer (Dangla-Pélissier et al., 2021). ClustAGE, a recently developed software program specifically designed to identify accessory genes within a bacterial species, was used to compare the accessory genome content of 14 *P. aeruginosa* strains, including PAO1 and PA14. Within the 14-strain set, 0.5% of PAO1 sub-element sequences were uniquely found in PAO1. Low variability in the PAO1 genome is ideal, as it is the most widely used reference strain for the species. A more significant 7% percent of sub-element sequences were unique to only PA14 (Ozer, 2018).

**Figure 1:**
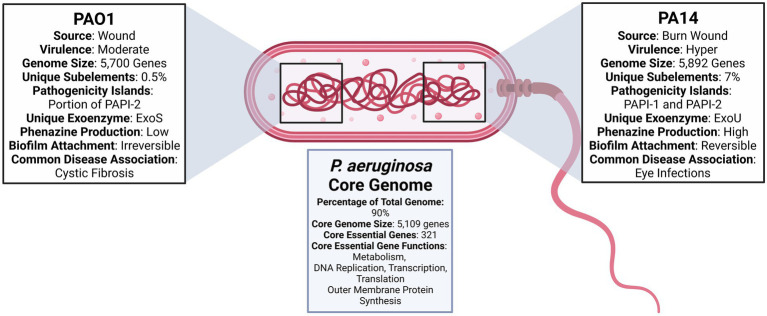
Diagram comparing *Pseudomonas aeruginosa* PAO1 and PA14 (Stover et al., 2000; He et al., 2004; Filloux, 2011; Mikkelsen et al., 2011; Ozer et al., 2014, 2019; Bosire et al., 2016; Mathee, 2018; Ozer, 2018; Chandler et al., 2019; Poulsen et al., 2019; Lee et al., 2020).

PA14 has over 800 genes not present in PAO1 that span 26 out of 28 functional categories. The only two categories with no PA14 specific genes are chemotaxis and quinolone signal response. An exhaustive list of PA14 specific genes can be found at http://pa14.mgh.harvard.edu/cgi-bin/pa14/annotation/start.cgi. Genomic and other comparisons between PAO1 and PA14 are summarized in Figure 1. The question that remains is whether accessory genes can affect essential gene pathways and alter the efficacy of therapeutics designed to target the essential core genome. Genes acquired through horizontal gene transfer are known to affect intercellular interactions and to induce system-level effects (McInerney et al., 2017). Therefore, opportunities exist to rigorously evaluate the effect of accessory genes on the essential core genome while accounting for infection source and environmental composition. Such experimental designs would aid in determining the need for targeting accessory genes and essential core genes.

### Genomic sequencing

The genome of PAO1 was fully sequenced in 2000 and is now known to be 6.3 Mbp (66.6% G + C content) in size, with 5,700 genes. Of these, 5,584 are predicted open reading frames (ORFs; Stover et al., 2000; de Sousa et al., 2021). Sequencing of PAO1 revealed that *P. aeruginosa* has more regulatory genes than other sequenced bacteria genomes. The bacterium also has higher expression of transcriptional regulators in the LysR, AraC, ECF-σ, and two-component regulator families (Stover et al., 2000). In comparison to other bacterial genomes, PAO1 contains a substantially higher 150 genes encoding outer membrane proteins that have virulence, antibiotic, adhesion, and movement functions (de Sousa et al., 2021). With the exception of Pf1-like filamentous phage Pf4, PAO1 is absent of the prophages often identified in clinical strains (Diggle and Whiteley, 2020).

The genome of PA14 is slightly larger than that of PAO1, with 5,892 coding sequences (Ozer et al., 2014). The sequencing of PA14 in 2004 revealed a high level of similarity to the genomic sequence of PAO1 (He et al., 2004; Lee et al., 2006). As previously stated above, the most significant difference is the inclusion of two pathogenicity islands PAPI-1 and PAPI-2 in the genome of PA14. PAO1 only carries a portion of the PAPI-2 island and does not contain any portion of PAPI-1 (He et al., 2004). The PAPI-2 pathogenicity island contains the gene that produces exoU—a type III secreted protein involved in virulence (de Sousa et al., 2021). In addition to genetic sequencing, transcriptome analysis has identified gene categories likely to undergo variable expression in different environments. After culturing PA14 in 14 different conditions, Dotsch et al. identified 796 genes with high variable expression. The three genetic functional categories with the highest genetic variable expression were secreted factors (51%), noncoding RNA (approximately 50%), and chaperones and heat shock proteins (over 30%; Dötsch et al., 2015). These findings underscore secreted factors and stress response factors as key areas in which genetic expression may be skewed due to environmental differences.

## PAO1 and PA14 virulence determinant differences

The most prominent and frequently investigated differences between PAO1 and PA14 are in the area of virulence. PAO1 is commonly viewed as a moderately virulent strain and PA14 as a hypervirulent strain (Mikkelsen et al., 2011). One of the well-known additions to PA14’s accessory genome includes two pathogenicity islands PAPI-1 and PAPI-2 that have been identified as contributors to PA14’s increased virulence (Dangla-Pélissier et al., 2021). Additionally, a strain-specific mutation in *ladS* and the secretion of exoU toxin has also been found to contribute to PA14’s cytotoxicity and disease severity (Filloux, 2011; Mikkelsen et al., 2011; Foulkes et al., 2019). Other similarities and differences in PAO1 and PA14’s secretory factors, metabolic activity, chemotactic ability, and quorum sensing functions can shed light on the appropriateness of each reference strain for various applications.

### Secretory factors

Secreted factors, including proteases, toxins, and enzymes, are essential contributors to *P. aeruginosa’*s virulence (Azam and Khan, 2019). Nine secretion systems (T1SS through T9SS) have been identified in prokaryotes. Only five—T1SS, T2SS, T3SS, T5SS, and T6SS—pertain to *P. aeruginosa* to date (Azam and Khan, 2019). Bleves et al. have reviewed the protein secretion systems of *P. aeruginosa* in detail (Bleves et al., 2010). Recently, the type III secretion system (T3SS) has gained increased attention in relation to PAO1 and PA14 strains (Horna and Ruiz, 2021). T3SS is a complex syringe-type secretion system that injects effectors into host cells and alters host immune responses. A recent and detailed review of the system and the four effector proteins it secretes (Table 1) can be found here (Horna and Ruiz, 2021).

**Table 1:** T3SS effector proteins, associated reference strains, and protein functions (Filloux, 2011; Azimi et al., 2016).

Effector Protein	PAO1/PA14 Strain	Characteristics/Function
exoS	PAO1	GTPase activating protein (GAP) domain Adenosine diphosphate ribosyl transferase domain
exoT	PAO1 PA14	GTPase activating protein (GAP) domain Adenosine diphosphate ribosyl transferase domain
exoU	PA14	Phospholipase function Cytotoxic function
exoY	PAO1 PA14	Adenylate cyclase function

In regard to PAO1 and PA14 strains, two of the secreted effector proteins exoS and exoU were recently used as major delineators in a five division clade system of *P. aerguinosa* clinical and environmental isolates. Both strains secrete exoT and exoY, but only PA14 secretes exoU and only PAO1 secretes exoS (Filloux, 2011). With these two exoenzymes as primary differentiators, researchers found that 98 % of the isolates (over 700 strains in one set and over 1,000 strains in another set) closely aligned with either PAO1 or PA14 (Freschi et al., 2019; Ozer et al., 2019). Such a large percentage of isolates being closely related to PAO1 or PA14 further validates the clinical relevance of the two reference strains. For focus, this section will discuss recently identified differences in genetic and phenotypic expression of exoY and exoT, which are common to both PAO1 and PA14.

While both PAO1 and PA14 produce exoY, which has been found to inhibit host immune responses and to cause lung damage in *in vivo* models, the expression differs between the strains. Belyy et al. found that the PA14 exoY protein has an altered C terminus, containing a frame shift and amino acid substitutions, when compared to the exoY C terminus in PAO1. This mutation resulted in lower toxicity and activity in an *in vitro* yeast model. Such a mutation could be a fitness advantage, as the exoU protein secreted by PAO1 confers high toxicity and virulence to the strain (Belyy et al., 2018; Foulkes et al., 2019). Silistre et al. also found lower production of exoY from PA14 in comparison to PAO1 grown in LB culture media. Further, their findings that strains without exoY were more cytotoxic than strains with exoY support the notion that exoY reduce the toxic ability of other virulent factors. Silestre et al. observed a wide range of exoY activity in different strains. However, their findings may be skewed since a metal chelator was used to directly induce exoY expression in culture. In *in vivo* settings, bacteria must naturally come into contact with human cells to induce exoY expression. The *in vivo* environment may yield lower exoY expression than *in vitro*, where there are no barriers to prevent exoY expression (Silistre et al., 2021).

Regarding clinical expression, Gabrielaite et al. found that exoY expression is lost in the CF host environment (Gabrielaite et al., 2020). This correlates with observations that many virulence factors needed for acute infections are frequently selected against in the chronic infection state of CF (Winstanley et al., 2016). Although Gebrielaite et al. used clinical strains for their study, CF strains have been found to generally be PAO1-like, which increases the likelihood that similar observations should be made with PAO1 itself (Ozer et al., 2019).

Unlike the decreased expression of exoY *in vivo*, genes inducing PAO1 exoT expression increased nearly 30-fold in a mouse corneal infection model when compared to the expression level observed in *in vitro* biofilm (Yam et al., 2022). This corroborates evidence that T3SS factors are needed to establish infections. Since the PA14 phenotype has been categorized with strains that produce eye infections, a PA14 comparison study would further illuminate whether exoT activity follows a similar expression pattern among more virulent *P. aerguinosa* strains (Ozer et al., 2019). Overall, the T3SS system and its effector proteins have been instrumental in characterizing large numbers of *P. aerguinosa* strains from diverse origins and stages of infection. The resulting clade system will hopefully simplify understanding of the bacterium and paths to treatment.

### Nutrient intake and metabolism

The cytoplasmic nutrient intake system of *P. aeruginosa* also contributes to its virulence and with estimates between 200 and 427 membrane transporter genes (Stover et al., 2000; Johnson et al., 2008). The sequencing of PAO1 disclosed a large capacity for carboxylates, including four TRAP-T type dicarboxylate permeases, but a low capacity for sugar transporters (Stover et al., 2000). *Pseudomonas aeruginosa* only intakes fructose and N-acetylglucosamine through two sugar transporters involved in the phosphotransferase system (PTS). However, the organism has more Β-oxidation encoding genes, known and putative, than any other fully sequenced bacterial genome (Stover et al., 2000). Membrane transporters for PAO1 align very closely with those of PA14 with two exceptions –PA14 contains additional transporters for iron and nickel (Johnson et al., 2008). These genes assist in facilitating the metabolism of a wide range of carbon and nitrogen sources, contributing to the bacterium’s metabolic versatility.

Bartell et al. recently reconstructed the metabolic networks for PAO1and PA14 with the goal of identifying connections between the bacterium’s metabolism and virulence expression (Bartell et al., 2017). Upon assessing 90 carbon sources for growth utilization, 32 sources resulted in growth, experimentally and computationally in both strains. Glycine, adenosine, and DL, α-Glycerol-Phosphate yielded experimental and computational growth in PA14 but did not yield experimental growth for PAO1. Inosine yielded experimental and computation growth for PAO1, but experimental growth was not accomplished in the PA14 strain. PAO1 successfully grew on cis—Aconitic acid and Urocanic acid substrates, but it is unclear if these sources were tested with PA14 (Bartell et al., 2017).

By linking genes essential for growth to the production of virulence factors, the same investigators uncovered 43 metabolically essential genes integrated into the production of at least one virulence factor— including alginate, lipid A, and pyocyanin— in synthetic cystic fibrosis medium (SCFM). Overall, the *in silico* test linked the production of 17 virulence factors to genes essential for PA14 growth (Bartell et al., 2017). A similar comparison of genes and virulence factors for PAO1 would be beneficial in assessing if the same links are dependable targets in the less virulent strain. *In silico* experiments provide valuable starting points for potential therapeutic targets against virulence and growth, but experimental validation still remains a critical step.

The specific microbial environment, such as laboratory media or *in vivo* settings, has been found to alter genetic expression and gene essentiality, and metabolic activity (Frimmersdorf et al., 2010; Poulsen et al., 2019; Manzo et al., 2021). Additionally, variation in metabolite production has been found between *P. aeruginosa* strains (Purcaro et al., 2019). Studies have shown, however, that variations in the bacteria environment produced greater metabolic heterogeneity than are produced by strain variation (Mielko et al., 2019). A significant environmental consideration for *P. aerguinosa* metabolic studies is whether the environment is aerobic or anaerobic, or a combination of both. *P. aeruginosa* generally has the metabolic flexibility to rely on nitrate sources under anoxic conditions (Arai, 2011). This switch from an aerobic to anaerobic environment, as seen in the CF lung environment not only introduces metabolic changes but also changes in virulence factors. Such changes can alter antibiotic susceptibility and biofilm fitness (La Rosa et al., 2019; Schiessl et al., 2019; Jo et al., 2020).

Schiessl and colleagues observed that the production of phenazine, redox compounds active in *P. aerguinosa* virulence function and electron transport, provided carbon source dependent protection against antibiotics for *P. aeruginosa* strain PA14 (Bosire et al., 2016; Schiessl et al., 2019). Antibiotic tolerance was observed with glucose as the carbon source, which was diminished with a switch to succinate. Jo et al. also reported that the benefit of phenazine to PA14 survival was carbon source dependent (Jo et al., 2020). Studies have shown that using antibiotics in conjunction with specific carbon sources can effectively reduce bacterial antibiotic tolerance (Crabbé et al., 2019). PA14 is beneficial in such investigations since it is a more effective phenazine producer than PAO1 (Bosire et al., 2016). However, the CF lung environment typically consists of mucoid and non-mucoid strains, and *P. aeruginosa* biofilms contain cells with varying metabolic profiles. To mimic clinical conditions more closely, mucoid strains could be evaluated in co-culture with PA14 and or PAO1 in future CF investigations (Malhotra et al., 2018). Moreover, culture media that promotes anaerobic metabolism such as Mueller Hinton Broth (MHB), Cation Adjusted-Mueller-Hinton Broth (CAMHB), Luria−Bertani (LB), and Tryptone Soya Broth (TSB) could be beneficial in anaerobic metabolic investigations (Manzo et al., 2021).

Examination of PAO1 metabolism in wound infections showed an increase in the expression of genes associated with poorly oxygenated environments. These findings suggest that the bacterium may also utilize aerobic and anaerobic metabolism in wound infections. However, the level of aerobic versus anaerobic metabolism was not clarified (Turner et al., 2014). In their model wound infections, the investigators also identified long chain fatty acids as a likely preferred carbon source for *P. aeruginosa*. The same group also determined that several metabolites, including amino acids (glutamate, phenylalanine, tyrosine, asparagine, and aspartate), purines, p-aminobenzoate (PABA), pantothenate (vitamin B5), pyridoxal phosphate (vitamin B6), and riboflavin (vitamin B2) are necessary for *P. aerguinosa* fitness in chronic and acute wound infections. Since the metabolites are required for fitness, they may be ideal therapeutic targets to combat *P. aeruginosa* wound infections (Turner et al., 2014).

The metabolic profiles of the more virulent burn wound isolate PA14 were recently assessed in a novel meat model to recreate *P. aerguinosa* wound infection. The advantages of this model were to leave intact the native microbial community, which represents the bacteria found in the normal human flora, to observe the behavior of added PA14 (Phan et al., 2020). This infection model successfully resulted in higher production of the *P. aeruginosa*-specific metabolic compounds acetophenone, 2-aminoacetophenone, 2-nonanone, and 2-butanone in the PA14 infected model (Phan et al., 2020). A potentially valuable addition would be to determine if actual burned meat alters the metabolic profile, as seen in this experiment.

### Chemosensory pathway

There are 26 ORFs in PAO1 that are linked to putative chemosensory transducer proteins. These proteins play a role in attracting and propelling the bacterium to nutrient sources, including glucose, inorganic phosphate, and amino acids (Stover et al., 2000). The ORFs contain five gene clusters that produce proteins for four distinct and functionally different chemosensory pathways detailed in Table 2 (Matilla et al., 2021). The chemosensory pathways have different functions, such as controlling twitching motility and modulating bis-(3′-5′)-cyclic dimeric guanosine monophosphate (c-di-GMP). However, the majority of *P. aeruginosa* chemoreceptors are believed to activate the F6 pathway, which is dedicated to chemotactic function (Matilla et al., 2021).

**Table 2:** *Pseudomonas aeruginosa* chemotaxis pathways, pathway receptors, and pathway functions (Matilla et al., 2021).

Chemotaxis Pathway	Chemoreceptor (PAO1)	Pathway Function
F6 (Che)	23 chemoreceptors: PA1251 PA1608 PA2561 (CtpH) PA2573 PA2920 PA4915 PA2654 (TIpQ) PA4307 (PctC) PA4309 (PctA) PA4310 (PctB) PA4633 PA1646 PA4844 (CtpL) PA5072 (McpK) PA2652 (CtpM) PA2788 (McpN) PA4520 PA2867 PA1561 (Aer) PA0180 (McpA) PA4290 PA1423 (BdlA) PA1930 (McpS)	chemotaxis
F7 (Che2)	McpB/Aer2	unknown
ACF (Wsp)	WspA	Modulates bis-(3′-5′)-cyclic dimeric guanosine monophosphate (c-di-GMP)
TFP (Chp)	PilJ	Twitching motility

Some comparative chemotactic studies have been performed using the PAO1 and PA14 strains. One study investigated the activity of PAO1 PA3348, which was determined to be a putative gene for CheR1. CheR1 is a chemotaxis protein methyltransferase that promotes chemotaxis toward amino acids (Schmidt et al., 2011). This study demonstrated that CheR1 in both PAO1 and PA14 was necessary for flagella-facilitated chemotaxis. A slight difference in the swim pattern of both strains was seen, but such a difference may not significantly impact the value of CheR1 as a potential therapeutic target. Furthermore, although the PAO1 PA3348 gene in this study only displayed a 31% amino acid similarity to the gene product positively identified as CheR1 in *E. coli,* the experiments performed validated the PA3348 gene’s necessity in chemotaxis and biofilm formation (Winsor et al., 2005). These findings agree with data available at http://pa14.mgh.harvard.edu/cgi-bin/pa14/annotation/start.cgi, which shows no strain-specific genes between PAO1 and PA14.

*In vitro* chemotaxis experimental designs using PAO1 and PA14 are now improved to replicate infection settings better. For example, Behroozian et al. developed a more biologically relevant experimental system that integrated CF-derived bronchial epithelial (CFBE) cells, representative of CF airway epithelia, into a microcapillary assay with PAO1. This design was used to determine if bile was a significant chemoattractant for *P. aeruginosa*. The investigators demonstrated PAO1 chemotaxis to bovine bile, as in the traditional microcapillary chemotaxis design (Behroozian et al., 2022). Such as system has the potential to observe chemotaxis, binding, and colonization activity simultaneously. It would also be beneficial to determine if the genetic and phenotypic profile of PAO1 in the presence of both cells and chemoattractants is more in line with actual infection profiles, as opposed to the expression profile of *P. aeruginosa* in the presence of chemoattractants alone.

### Quorum sensing

There are key differences between PA14 and PAO1 quorum sensing (QS) mechanisms, which, in part, regulate the expression of virulence genes in a bacterial cell density-dependent manner (Lee and Zhang, 2015; Sana et al., 2019). Generally, QS in *P. aerguinosa* is governed by three systems—Las, Rhl, and Pqs that have been elegantly reviewed (Chadha et al., 2022). To identify a relationship between QS regulators and the level of virulence, investigators determined whether depletion of QslA protein, a QS deactivator, contributed to the hypervirulent phenotype of PA14. High levels of QslA protein were produced in the lower virulence PAO1 strain, whereas the QslA protein was minimally expressed in the hypervirulent PA14 strain. Deleting the *qslA* gene from PAO1 resulted in QS gene expression rates that were higher than those of PA14 (Sana et al., 2019). Notably, PAO1 and PA14 strains were grown in the traditional lysogeny broth (LB) media. QS is known to change at different stages of infections, which would impact virulence expression. If QslA is considered a virulent marker, evaluating QslA expression in more clinically relevant media, such as SCFM or serum-containing media, would be beneficial.

Other studies have simulated bacteremia conditions by growing PAO1 and PA14 in blood to determine if blood affects QS and virulence gene expression (Beasley et al., 2020). Overall, growth in the blood reduced the expression of some QS genes. Compared to growth in laboratory medium Luria-Bertani broth, growth of PAO1 in whole blood from healthy subjects revealed reduced expression of QS-related genes *lasB*, *phzB1*, *phzB2*, *rhlA*, and *rhlB* by over 85-fold (Beasley et al., 2020). QS-regulated genes *lasA*, *rhlA*, and *rhlB* expression in the PA14 strain significantly decreased at least 10-fold when comparing the expression levels in blood from healthy individuals and blood from individuals with severe burn injuries (Kruczek et al., 2016). The reduced gene expression could suggest that QS may not be critical for establishing infection. Moreover, the data further highlight the need to use clinically relevant media in infection experiments to compile clinically accurate data (Poulsen et al., 2019; Manzo et al., 2021).

Another experiment showed that reference strains successfully mimic QS activity of clinical strains when placed under the same conditions. Sotos-aceves et al. grew PAO1 in low phosphate (FDS) media to mimic the low phosphate environment present at the beginning of infection in those who have undergone chemotherapy or surgical procedures. The authors determined that phenazine pyocyanin production increased under low phosphate conditions, independent of the Las system. Las independent QS activation in PAO1 correlated with findings in other reference strains, including PA14 and other isolates (Soto-Aceves et al., 2021).

Whereas PAO1 and PA14 have successfully mimicked QS aspects, some strain genetic and phenotypic inconsistencies have been observed. Some examples are *MexT*, a significant QS factor and fitness regulator, and *LasR* found in PAO1 strains from different laboratories (Liu et al., 2022). Their observed mutations affected QS signal, virulence factor, biofilm formation, and motility expression. Only the production of pyoverdine, a primary *P. aerguinosa* siderophore and contributor to infection establishment, was consistent between the five PAO1 strains analyzed. Also mexT, has been posited as a PAO1 strain integrity indicator due to its high potential for mutation (LoVullo and Schweizer, 2020). Although it is crucial to use reference strains as close to the sequenced version as possible, *mexT* and *lasR* are frequently mutated in clinical isolates. This raises the question of whether the strain variation for these genes is a consequence of inconsistent maintenance methods or internal bacterial activity.

Laboratory and clinical conditions both produce *MexT* and *LasR* mutations, but their respective results diverge in the area of social cheating and policing. PAO1 and PA14 have been used to investigate “social cheating” *in vitro* whereby a single carbon source, such as casein, is used to feed wild-type (WT) and mutant strains (e.g., *lasR* and *pvdS* mutants frequently found in CF isolates; Özkaya et al., 2018). The mutant strains are expected to “cheat” by utilizing proteases produced by WT strains (Chen et al., 2019). In a biofilm or liquid media culture, these two strain types can coexist unless the mutant strain(s) outgrow the WT and cause the population to collapse. Moreover, the “cooperator,” or WT strain is also thought to produce cyanide to “police” mutant cheating strains in an effort to prevent mutant strain overgrowth and subsequent collapse (Castañeda-Tamez et al., 2018; Yan et al., 2018).

Albeit the above observations are consistent *in vitro*, they are difficult to recreate or observe *in vivo*. Investigators have observed clinical bacterial social interactions that suggest a cooperator-cheat dynamic, but the certainty of this phenomenon is debatable (Rezzoagli et al., 2019). The infection environment has many carbon and nutrient sources as opposed to one, which is typically used in laboratory social cheating experiments. Also, the microbial community is heterogeneous, which introduces many additional factors, resources, and competition aspects to the environment. Rezzoagli et al. infected the *Caenorhabditis elegans* nematode model with PAO1 to determine if a siderophore-double mutant and Las-deficient mutant could outcompete the WT PAO1. Though the mutants seemed to benefit from the WT strain, they could not outcompete or displace it (Rezzoagli et al., 2019).

One critical difference between the laboratory media culture and human infection environment is the spatial structure of the infection environment and bacterial community. Such structure is not present in a laboratory media culture. Mund et al. added agar to the culture media for spatial and structural arrangement in their PAO1 and *lasl* mutant social cheating experiment. This addition significantly reduced the *lasl* mutant fitness and, therefore, the social cheating. Agar prevented free diffusion of signal molecules, which would not be present in an *in vivo* environment (Mund et al., 2017). Differences in these *in vivo* and *in vitro* social cheating observations underscore the importance of *in vitro* experiments mimicking *in vivo* environments as closely as possible.

### Biofilm formation

The tendency of *P. aeruginosa* to cause persistent infections with increased mortality rates is partly due to its ability to readily form biofilms (Thi et al., 2020). Biofilm formation allows the bacterium to circumvent host immunity and increase antibiotic-resistance (Thi et al., 2020). A number of studies have revealed genetic and phenotypic similarities and differences in PAO1 and PA14 biofilm formation. One key contributor to biofilm persistence in chronic infections is the development of the rugose small colony variants (RSCVs) phenotype (Pestrak et al., 2018). In PA14 infected burn wounds, wrinkly spreader phenotype (Wsp) chemoreceptor wspA and methylesterase wspF both underwent mutations to yield RSCVs that favored biofilm formation (Matilla et al., 2021). RSCVs form colonies with a wrinkled appearance, are more prone to aggregation and surface attachment, and exhibit increased exopolysaccharide (EPS) production (Starkey et al., 2009). RSCVs are associated with hyper-biofilm formation and tend to characterize poorer clinical outcomes as repeatedly seen in chronic infections (Marshall et al., 2021). Some studies also suggest that the appearance of RSCVs may be a response to extended antibiotic therapy (Starkey et al., 2009). A study found that RSCVs accounted for 30% of PA14 antibiotic-resistant colonies and also formed among PAO1 populations (Drenkard and Ausubel, 2002; Pu et al., 2018; Marshall et al., 2021).

Both strains have also been genetically modified to express the RSCV phenotype, the subsequent hyper-biofilm formation was used to identify underlying mechanisms and immune responses. An RSCV PAO1 strain, PAO1Δ*wspF* increased severity and prolonged infection in both porcine burn wound and pulmonary infection models (Pestrak et al., 2018). Infection with PAO1Δ*wspF* also stimulated a robust immune response that resulted in tissue damage (Pestrak et al., 2018). With PA14, other investigators observed that inactivation of a serine hydroxymethyltransferase (SHMT) *shrA* PA2444 resulted in conversion to the RSCV phenotype (Pu et al., 2018). The SHMT enzyme traditionally involved in metabolism was observed to control rugose colony formation by regulating cyclic diguanylate (c-di-GMP), which is elevated in biofilms (Pu et al., 2018; Thi et al., 2020). In PAO1, *shrA* was identified as a QS-induced gene and, in both strains, SHMT *shrA* demonstrated significant involvement in the cell community functions that lead to biofilm formation (Schuster et al., 2003). The genetic alteration of PAO1 and PA14 strains to express an RSCV phenotype presents another means of improving the clinical relevance of these widely available reference strains.

There are several methods to elicit the RSCV phenotype in *P. aeruginosa* laboratory strains. However, it is important to note that experiments involving cultures that consist solely of RSCVs may produce heightened responses that would not be seen in clinical settings. Chronic infections in which RSCVs develop can also consist of other small colony variants (SCVs) and other phenotypic variations (Pestrak et al., 2018). A study found that 3% of colonies from CF sputum samples were SCVs and of the *P. aeruginosa* isolates examined; an average of 2.2 morphotypes per sample were identified (Haussler et al., 1999). These findings confirm that RSCVs would be found in conjunction with other *P. aeruginosa* phenotypes in clinical samples.

Although PAO1 and PA14 have similar colony appearances, there are key differences in their attachment and biofilm formation strategies (Lee et al., 2020). PAO1 was found to quickly attach to surfaces and progress to biofilm formation in an irreversible process. PA14 however, displayed less initial commitment to surface attachment—detaching from surfaces and forming memory of attachment behavior that was then passed to future PA14 generations. Such behavior yields multiple PA14 generations that are adept biofilm developers. There are mechanistic reasons for the differences in PAO1 and PA14 surface attachment strategies. The Wsp pathway mediates PAO1’s surface recognition strategy, which results in higher production of exopolysaccharides that promotes attachment of nearby bacterial cells. PA14’s surface recognition strategy, however, is driven by the Chp pathway, which adjusts cAMP concentrations to promote planktonic cell surface memory (Lee et al., 2020; Matilla et al., 2021).

The behaviors mentioned above should apply to many *P. aeruginosa* strains, as most strains are either PAO1-or PA14-like (Ozer et al., 2019). However, *in vivo* biofilms are far more complex than those grown in laboratory media. For example, biofilms isolated from CF patients had a “sponge-like” appearance with mucus or lung fluid punctuating masses of bacterial cells. Conversely, *in vitro* biofilms were more homogenous with dense or mushroom-like structures (Roberts et al., 2015; Kragh et al., 2019). To address the structural and metabolic discord between the two environments, Harrington et al. optimized an *ex vivo* pig lung model (EVPL) with PA14 and ASM (Artificial Sputum Media) for more accurate *P. aerguinosa* biofilm formation. While purine biosynthesis appeared to be essential *in vitro* in the presence of ASM2 but not in actual CF sputum, the addition of the media to the EVPL model removed this discrepancy. Additionally, the researchers confirmed that PA14 used the expected Pel polysaccharide and GacAS regulatory system pathways to produce biofilms structurally similar to those in CF patients (Harrington et al., 2020). A comparative study with PAO1 would be ideal, as most CF strains tend to be PAO1-like (Ozer et al., 2019). The initial achievements of this model should assist in reducing discrepancies between findings in future *in vitro* biofilm experiments and human infection.

### Immunity

*Pseudomonas aeruginosa* generally possesses a number of factors that antagonize host immunity. Flagella and LPS are two antagonists to innate immune system receptors TLR5 and TLR4, respectively (Lavoie et al., 2011). Unfortunately, *P. aeruginosa* flagella and LPS in clinical and laboratory strains are prone to mutations or deletions, which can dampen or alter the innate immune response to initial infection. When exposed to sputum, *P. aerguinosa* downregulates flagellin and flagella (Lavoie et al., 2011). If this is the clinical expectation, pre-clinical studies investigating these factors could investigate whether the same factors are downregulated in the SCFM that would be used *in vitro*. Additionally, pyocyanin and rhamnolipid-producing strains, like PAO1 and PA14, can induce neutrophil cell death and express the molecules more potently in the bacteria’s stationary or high-density growth phases, the latter of which correlates to biofilm formation (Lavoie et al., 2011; Das and Manefield, 2012). Both PAO1 and PA14 have been investigated for their ability to overcome or circumvent host immunity to establish infection.

LasB, a prominent virulence factor and metalloprotease identified in PAO1, have been shown to degrade interleukin 6 (IL-6), an early immune responder to infection, and trappin-2, an antimicrobial molecule secreted from epithelial cells, *in vitro* and *in vivo* (Tanaka et al., 2014; Saint-Criq et al., 2018). LasB also demonstrated the ability to degrade pulmonary surfactant protein-A (SP-A), which binds to and aggregates bacteria for phagocytosis *in vivo* (Kuang et al., 2011). LasB downregulated the IL-6/STAT3 signaling pathway, which interfered with tissue repair. However, overexpressing IL-6 and trappin-2 *in vivo* could prevent death from PAO1 infection (Saint-Criq et al., 2018). In addition to these findings, it could be beneficial to determine if overexpression of IL-6 and trappin-2 would have the same effect in a more virulent LasB-expressing strain such as PA14 (Winsor et al., 2016).

Within gram-negative bacteria species, LPS is a prominent inducer of inflammation and of innate immunity (Ciornei et al., 2010). While PAO1 and PA14 were found to have similar S-form LPS, changes in the lipid-A structure of PAO1 in biofilm formation were found to stimulate a hyperinflammatory response in human and murine macrophages *in vitro* (Ciornei et al., 2010; Wang et al., 2021). The inflammatory response was specific to PAO1 in biofilm formation and was significantly higher than that caused by planktonic PAO1 (Wang et al., 2021). *In vivo* studies could shed additional light on whether other factors contribute to the level of the inflammatory response to LPS in biofilm formation. Also, mutations in the *wbpX* and *migA* genes in PA14 have contributed to differences in PA14 LPS chain length, O-specific antigen (OSA) serotypes, and common polysaccharide antigen (CPA; Hao et al., 2015). These deviations from those seen in PAO1 are additional areas that can be investigated for the impact on host inflammatory responses (Hao et al., 2015).

In addition to defenses against inflammation, *P. aeruginosa* has protective mechanisms against reactive oxygen species (ROS), which aid in bacterial engulfment and killing (da Cruz Nizer et al., 2021). PA14 response to antibacterial agents and hypohalous acids—hypochlorous acid (HOCl), a bleach component, and hypothiocyanous acid (HOSCN)—was investigated in an *in vitro* study (Farrant et al., 2020). In response to the acids, PA14 produced polyphosphate to defend against the protein aggregation the hypohalous acids induced and to activate the transcriptional regulator RclR system, which increased pyocyanin production and downregulated chaperone genes (Groitl et al., 2017; Farrant et al., 2020). The RclR system and the polyphosphate chaperone are highly conserved and would theoretically show similar activity in PAO1 (Groitl et al., 2017; da Cruz Nizer et al., 2020). Since LB was used to grow the bacteria in this study, a comparison study with SCFM would be beneficial to further validate the results, as CF was the condition of interest.

## PAO1 and PA14 problems in treating infections

### Antibiotic resistance

Designated as one of the ESKAPE pathogens by the WHO, *P. aeruginosa*’s MDR nature has made it a priority for the development of new therapeutics (De Oliveira et al., 2020). As previously mentioned, *P. aeruginosa* has an abnormally high number of genes (150) that produce outer membrane proteins (OMPs), which contribute to its survival by exporting antibiotics and virulence factors and providing structural support to motility and adhesion factors. Additionally, 12 resistance-nodulation-cell division (RND) efflux pumps further enhance resistance to antibiotic and environmental stressors with the *mex*AB-*opr*M, *mex*CD-*opr*J, *mex*EF-*opr*N, and *mex*XY (−*opr*A) pumps facilitating the majority of resistance (Dreier and Ruggerone, 2015). The characteristically low permeability of the *P. aeruginosa* membrane also contributes to intrinsic antibiotic defense (Chevalier et al., 2017; Coleman et al., 2020). These factors are highly conserved among *P. aeruginosa* strains, and are found in both PAO1 and PA14 (Valot et al., 2015). Experimental environments for antimicrobial testing are especially important to consider, as *P. aeruginosa* resistance increases in actual human infections (Cornforth et al., 2018).

In addition to the core resistance mechanisms of *P. aeruginosa*, studies with PAO1 and PA14 have identified other potential mechanisms that may contribute to antibiotic resistance. In one study, efflux pump MexXY-OprM was primarily responsible for PAO1 resistance to tigecycline, a minoclycline analog. However, mutants lacking the MexXY-OprM pump and exposed to tigecycline produced subsequent mutants resistant to tigecycline. These occurrences suggest that other efflux pumps can compensate for the lack of MexXY-OprM (Dean et al., 2003). Another study used PAO1 to explore the resistance potential of *P. aeruginosa* to prolonged exposure to clindamycin/rifampicin-impregnated catheters (CR-IC). PAO1 demonstrated increased survival in comparison to two *S. aureus* strains exposed to CR-IC. Fourteen porin, efflux pump, translation, and transcription antibacterial resistance proteins were upregulated in CR-IC treated planktonic cells. However, the CR-IC treatment did not significantly alter the minimum inhibitory concentrations (MIC) of tested antibiotics during the 144-h exposure time (Sung et al., 2021). Further studies could reveal whether these proteomic changes impact antibiotic-resistance over longer periods of time.

As *P. aeruginosa* is known to switch phenotypes in different stages of infection, one study looked at the resistance of PA14 to a variety of antibiotics during the swarming stage. PA14 would likely display swarming motility during early infection, as the organism tends to progress toward a non-motile phenotype in later stages of infection (Winstanley et al., 2016; Coleman et al., 2020). Swarming PA14 showed increased resistance to aminoglycosides (amikacin, kanamycin, gentamicin, and tobramycin), to—lactams (ceftazidime, meropenem, and piperacillin), macrolides (erythromycin and azithromycin), chloramphenicol, tetracycline, ciprofloxacin, and trimethoprim (Coleman et al., 2020). In the case of tobramycin resistance, reduced expression of *wzz*, *wbpI, wbpA*, and *wbpL* may be contributing factors, as well as increased expression of multidrug efflux pump MexXY (Coleman et al., 2020). Reduced expression of ribosomal proteins might possibly contribute to resistance in antibiotics (aminoglycosides, chloramphenicol, macrolides, and tetracycline) that target ribosomal structures (Coleman et al., 2020). These results were derived from *in vitro* experiments, in which swarming activity was monitored in agar concentrations that allowed for motility.

Another study identified resistance genes for PA14 in non-motile biofilm formation (Zhang et al., 2013). PA0756–0757 (a possible two-component regulatory system protein), PA2070 (a predicted type I export signal), and PA5033 (a predicted member of the TonB-dependent heme/hemoglobin receptor family) all demonstrated increased expression and suggested contribution to antibiotic resistance. These genes were also tested *in vivo* and were concluded to contribute to *Caenorhabditis elegans* fatality (Zhang et al., 2013).

The survival cost of the flagellar structure of PA14 in the presence of antibiotics, specifically vancomycin, has also been evaluated *in vitro* (Rundell et al., 2020). PA14 appeared to favor a form without the flagellar structure in the presence of vancomycin, but the mechanism driving this survival conformation remains unclear (Rundell et al., 2020). However, QS regulators LasR, RhlR, and PqsR were determined to contribute to fitness during antibiotic treatment (Rundell et al., 2020).

In PA14, *P. aeruginosa*-produced antibiotic pyocyanin has demonstrated the ability to cause increased resistance to antibiotics fluoroquinolones, chloramphenicol, and trimethoprim/sulfamethoxazole as a side effect of the mechanisms used to protect itself against the natural antibiotic it produced (Meirelles et al., 2021). The study by Meirelles et al. raised interesting considerations for *in vivo* implementation of *in vitro* tested antimicrobials. Natural antibiotics such as pyocyanin may contribute to differences seen between MIC *in vitro* and *in vivo*, as pyocyanin is typically not expressed in the low concentrations used to test antimicrobials *in vitro*. This lack of presence or low expression *in vitro* may allow antimicrobial susceptibility that a higher expression *in vivo* would prevent (Meirelles et al., 2021).

Another study by Migliorini et al. shed additional light on the differing effects of antibiotics on laboratory and clinical *P. aeruginosa* strains (Migliorini et al., 2019). Sub-lethal concentrations of ciprofloxacin caused mutagenesis 10 times the baseline in PAO1 and PA14. However, only a 3-fold increase in mutagenesis was seen in clinical strains subjected to sub-lethal ciprofloxacin concentrations. The sub-lethal dose of ciprofloxacin led to an increased optical density (OD) in PA14 compared to clinical strains. Such result suggests reduced replication of PA14 or increased mortality among clinical isolates. This deviation in strain behavior could be caused by the clinical strain production of antimicrobial bacteriocin Pyocin S2 that was not present in PA14 (Migliorini et al., 2019). The production of pyocin S2 may prevent mutagenesis in the producer strains. Additionally, transitioning clinical strains from an *in vivo* environment to laboratory media can alter genetic expression significantly (Migliorini et al., 2019).

The knowledge conferred from these studies can aid in novel gene target identification to kill or restore antibiotic susceptibility in *P. aeruginosa*, and aid in future experimental designs to accommodate the limitations of *in vitro* studies.

### Quorum quenching

In light of the multi-drug resistance in *P. aeruginosa*, the discovery of novel alternative and adjunct therapeutics has become a priority. Quorum quenching, the use of quorum inhibitors to disrupt QS, is one area under investigation as potential adjunct antibiotic therapy (Hemmati et al., 2020). One advantage to quorum quenching is that it inhibits QS without killing the bacteria. This therapeutic approach does not pressure the bacteria to develop resistance against the inhibitors, thus permitting the host immune system to clear the infection (Hemmati et al., 2020).

A large number of QS inhibitors (QSI) have been naturally and synthetically derived and demonstrated efficacy in preventing or disrupting biofilm formation by both PAO1 and PA14 (Scoffone et al., 2019; Hemmati et al., 2020). QSIs have been isolated from bacteria such as *Vibrio alginolyticus* and the QS inhibiting metabolites from *V. alginolyticus* prevented PAO1 biofilm formation and reduced the expression of virulence factors involved in motility, rhamnolipid biogenesis, and elastase activity (Song et al., 2018; Hemmati et al., 2020). Another natural QSI proanthocyanidins (cerPAC), derived from cranberry extract, downregulated QS *lasIR* and *rhlIR* genes and reduced virulence factors to prevent lethal *P. aeruginosa* PA14 infection in *Drosophila melanogaster* (Maisuria et al., 2016; Scoffone et al., 2019). Metal complexes such as copper (II) sulfate pentahydrate-curcumin complex (Cu-CUR) were also found to have QS inhibitory properties in PAO1 by reducing *lasI* and *lasR* gene expression, biofilm formation, pyocyanin, and alginate production, motility, and increasing sensitivity to H_2_O_2_ (Gholami et al., 2020).

Any naturally or synthetically derived compound that prevents the production of or degrades N-Acyl homoserine lactone (AHL), reduces AHL synthase activity, or antagonizes other QS signaling molecules would be potential adjunct candidates for current antimicrobials (Hemmati et al., 2020). However, as previously stated, *P. aeruginosa* QS is significantly reduced in clinically relevant media, which correlates with the lower QS activity observed in human infection (Cornforth et al., 2018; Beasley et al., 2020). A clinically relevant environment is critical to producing reliable interactions between *P. aeruginosa* strains and therapeutics.

### Antimicrobial peptides

Antimicrobial peptides (AMPs), which are characteristically short and positively charged, have been widely tested alternatives to antibiotic resistance. Naturally produced by living organisms or synthetically designed, AMPs have demonstrated broad efficacy against pathogens with the reduced potential to cause antimicrobial resistance (Mulani et al., 2019). Numerous studies have described AMPs with bactericidal activity against *P. aeruginosa*. Synthetic peptide 6 K-F17 (sequence: KKKKKK-AAFAAWAAFAA-NH2) demonstrated activity against MDR *P. aeruginosa* and PAO1 strains (Beaudoin et al., 2018). Moreso, this peptide successfully decreased PAO1 and MDR *P. aeruginosa* biofilms *in vitro*, while proving to be non-toxic to human bronchial epithelial cells and non-hemolytic to human erythrocytes (Beaudoin et al., 2018). Another synthetic peptide 1018 inhibited the growth of PA14 planktonic cells at an MIC of 64 μg/ml and reduced swarming motility by 95% at a concentration of 1.0 μg/ml *in vitro* (Wilkinson et al., 2021). These results show the potential for peptide 1080 to impact the early stages of *P. aeruginosa* infection. Human β-defensin 3 combined with a carbohydrate-binding domain was efficacious against PA14 *in vitro,* reduced biofilm formation, and production of factors of T3SS, including pyocyanin, exoU, exoS, and lasB (Wu et al., 2020). Another synthetic AMP-dendrimer G3KL, reportedly killed PA14 faster than polymyxin B without producing resistant mutants (Ben Jeddou et al., 2020). These results are encouraging, given that polymyxins such as colistin have significant toxicity and tend to lead to resistance in *P. aeruginosa*. Dendrimers may be promising alternatives as another dendrimer T7 tested in this study had killing activity against polymyxin-resistant mutants (Ben Jeddou et al., 2020).

Many AMPs have demonstrated activity against *P. aeruginosa*, but hurdles to clinical application remain. Protease degradation, possible mammalian cytotoxicity, deleterious local or systemic effects, production expense, gut flora changes, and loss of potency in decreased salt concentrations or with plasma proteins are among the *in vivo* challenges of AMPs (Mulani et al., 2019; Wu et al., 2020). Research that underscores AMP structural modifications, encapsulation or other delivery methods, and synergistic antibiotic applications is a key to overcoming these challenges (Mulani et al., 2019). In addition to the challenges presented by AMP composition are the challenges presented by the *in vitro* testing environment. The currently recommended media for antimicrobial susceptibility testing (AST) is Mueller-Hinton broth (MHB). However, there have recently been calls to update AST testing media to ones that are more representative of the biological environment and do not contain components that unnecessarily interfere with AMP activity (Nizet, 2017; Mercer et al., 2020).

### Mutations in strains and implications

While many genomic, *in silico* and laboratory studies have built genetic identities for PAO1 and PA14, environmental factors can affect their genetic expression. Factors such as laboratory media conditions and clinical isolation can alter gene expression, which could affect the therapeutic value of potential targets. For example, genes selected as therapeutic targets because they were observed to be essential to the bacterium’s survival, were later redefined as “conditionally essential” under specific growth conditions. Genes that exhibited essential characteristics *in vitro* were not necessarily essential *in vivo* (Poulsen et al., 2019). Also, genetic expression and bacterial behaviors are apt to change if *P. aeruginosa* is moved from an infectious environment into laboratory conditions. Such changes can skew results as bacteria switch survival mechanisms from an *in vivo* to *in vitro* atmosphere (Migliorini et al., 2019). In comparison to freshly isolated clinical strains, laboratory-maintained strains PAO1 and PA14 have also demonstrated a lower ability to form biofilms and switch from non-mucoid to mucoid phenotypes (Migliorini et al., 2019).

PAO1 and PA14 can acquire mutations through subculturing and storage practices (Chandler et al., 2019; Migliorini et al., 2019). A genomic and phenotypic comparison study was performed on 10 PAO1 strains from different laboratories (Chandler et al., 2019) and the results showed that the strains maintained comparable planktonic and biofilm growth, antibiotic susceptibility, and immunostimulatory profiles. However, notable differences were observed among secreted molecules involved in virulence and infection, such as rhamnolipid, pyoverdine, exopolysaccharide, Pseudomonasquinolone signal (PQS), pyocyanin, and outer membrane vesicles (Chandler et al., 2019). Such variations can alter the findings of subsequent *in vivo* studies (Chandler et al., 2019).

Reducing the number of subcultures, identifying the culture lineage and genetically sequencing strains can address some of these laboratory challenges. These practices can support the efforts to provide quality controlled and reproducible results (Chandler et al., 2019).

## Concluding remarks

Reference strains PAO1 and PA14 are critical to *P. aeruginosa* research, as they are easily accessible, fully sequenced, and representative of moderate virulence and hyper virulence, respectively (Winsor et al., 2016). The recent development of the five clade system used to categorize *P. aerguinosa* strains confirmed that 98% of strains are either PAO1-or PA14-like. The availability of PAO1 and PA14 genomes have been beneficial in identifying typical and diverging traits in metabolism, protein secretion, QS, biofilm formation, and antibiotic resistance (Winsor et al., 2016).

These reference strains have contributed substantially to novel therapeutic endeavors, which are crucial, as developed antibiotic resistance has made *P. aeruginosa* challenging to treat (De Oliveira et al., 2020). Knowledge of the mechanisms by which PAO1 and PA14 have developed resistance to various antibiotics has shed light on possible targets for future therapeutics. Targeting resistance mechanisms can restore sensitivity to traditional antibiotics that have already been evaluated for clinical efficacy and safety. Also, PAO1 and PA14 have been used to test novel QSIs, which ideally would prevent *P. aeruginosa* from forming difficult-to-treat biofilms and can be used in conjunction with traditional antibiotics. Methods to restore antibiotics sensitively are ideal, but novel therapeutics, such as AMPs, are also critical in expanding the arsenal of therapeutics against *P. aeruginosa*, as they are less likely to provoke resistance (Mulani et al., 2019). AMP experiments with PAO1 and PA14 have led to the identification of potential novel therapeutics that have demonstrated success in killing *P. aeruginosa* and reducing virulence in areas such as QS, secretion, and motility (Beaudoin et al., 2018; Mulani et al., 2019; Ben Jeddou et al., 2020; Wu et al., 2020; Wilkinson et al., 2021). However, it is important to note that the testing environment can be as important as the therapeutic itself. The more the testing environment mirrors the actual host environment, the more dependable *in vitro* results will be (Ersoy et al., 2017).

Experiments involving PAO1 and PA14 require some considerations to yield data that is as consistent and reproducible as possible. PAO1 and PA14 are prone to developing mutations that stem from varying growth and storage conditions (Chandler et al., 2019). Also, these strains can yield results that differ greatly in comparison to clinical strains transitioned from an infectious environment to laboratory media (Migliorini et al., 2019). Even in testing therapeutics, using different laboratory media can affect the therapeutic MICs and other results (Migliorini et al., 2019). Maintaining PAO1 and PA14 cultures in consistent environments, using low-passage cultures and genomic sequencing can reduce variations in accrued data (Chandler et al., 2019). Overall, PAO1 and PA14 are necessary and effective models in the quest to understand and treat a highly mutable and MDR bacterium.

## Author contributions

AG performed the literature search and wrote the manuscript. VD read and edited the manuscript and oversaw the compilation of the review. RS and DO read and edited the manuscript. All authors contributed to the article and approved the submitted version.

## Funding

This research was supported by NSF-HBCU-UP (HRD-1911660), NIH-NIGMS-RISE (1R25GM106995-01), NSF-HBCU-RISE (HRD-1646729) grants, and the Ph.D. program in Microbiology at Alabama State University.

## Conflict of interest

DO is the President and CEO of Owen Biosciences Inc.

The remaining authors declare that the research was conducted in the absence of any commercial or financial relationships that could be construed as a potential conflict of interest.

## Publisher’s note

All claims expressed in this article are solely those of the authors and do not necessarily represent those of their affiliated organizations, or those of the publisher, the editors and the reviewers. Any product that may be evaluated in this article, or claim that may be made by its manufacturer, is not guaranteed or endorsed by the publisher.
